# Automatic extraction of upper-limb kinematic activity using deep learning-based markerless tracking during deep brain stimulation implantation for Parkinson’s disease: A proof of concept study

**DOI:** 10.1371/journal.pone.0275490

**Published:** 2022-10-20

**Authors:** Sunderland Baker, Anand Tekriwal, Gidon Felsen, Elijah Christensen, Lisa Hirt, Steven G. Ojemann, Daniel R. Kramer, Drew S. Kern, John A. Thompson

**Affiliations:** 1 Department of Human Biology and Kinesiology, Colorado College, Colorado Springs, Colorado, United States of America; 2 Department of Neurosurgery, University of Colorado Anschutz Medical Campus, Aurora, Colorado, United States of America; 3 Department of Physiology and Biophysics, University of Colorado Anschutz Medical Campus, Aurora, Colorado, United States of America; 4 Neuroscience Graduate Program, University of Colorado Anschutz Medical Campus, Aurora, Colorado, United States of America; 5 Medical Scientist Training Program, University of Colorado Anschutz Medical Campus, Aurora, Colorado, United States of America; 6 Department of Neurology, University of Colorado Anschutz Medical Campus, Aurora, Colorado, United States of America; Vellore Institute of Technology: VIT University, INDIA

## Abstract

Optimal placement of deep brain stimulation (DBS) therapy for treating movement disorders routinely relies on intraoperative motor testing for target determination. However, in current practice, motor testing relies on subjective interpretation and correlation of motor and neural information. Recent advances in computer vision could improve assessment accuracy. We describe our application of deep learning-based computer vision to conduct markerless tracking for measuring motor behaviors of patients undergoing DBS surgery for the treatment of Parkinson’s disease. Video recordings were acquired during intraoperative kinematic testing (N = 5 patients), as part of standard of care for accurate implantation of the DBS electrode. Kinematic data were extracted from videos post-hoc using the Python-based computer vision suite DeepLabCut. Both manual and automated (80.00% accuracy) approaches were used to extract kinematic episodes from threshold derived kinematic fluctuations. Active motor epochs were compressed by modeling upper limb deflections with a parabolic fit. A semi-supervised classification model, support vector machine (SVM), trained on the parameters defined by the parabolic fit reliably predicted movement type. Across all cases, tracking was well calibrated (i.e., reprojection pixel errors 0.016–0.041; accuracies >95%). SVM predicted classification demonstrated high accuracy (85.70%) including for two common upper limb movements, arm chain pulls (92.30%) and hand clenches (76.20%), with accuracy validated using a leave-one-out process for each patient. These results demonstrate successful capture and categorization of motor behaviors critical for assessing the optimal brain target for DBS surgery. Conventional motor testing procedures have proven informative and contributory to targeting but have largely remained subjective and inaccessible to non-Western and rural DBS centers with limited resources. This approach could automate the process and improve accuracy for neuro-motor mapping, to improve surgical targeting, optimize DBS therapy, provide accessible avenues for neuro-motor mapping and DBS implantation, and advance our understanding of the function of different brain areas.

## Introduction

Neurodegenerative disorders such as Parkinson’s disease (PD) are prevalent, affecting around 1.6% of the population [1]. Deep brain stimulation (DBS) is a well-established treatment of PD, targeting its motor, non-motor, and quality-of life implications. Treatment efficacy is dependent in part on optimal electrode placement [2–6]. Implantation into the subthalamic nucleus (STN) or globus pallidus internus (GPi), depending on the intended target, is aided by performing microelectrode recordings (MER) and evaluating kinesthetic responses [2–4, 6–9]. Awake protocols are generally advantageous, as patients exhibit improved outcomes given the ability to make decisions on electrode placement in the operating room with feedback [7, 10]. While this method for gauging motor relationships is effective [8, 11], it is accomplished through the subjective assessment of a trained clinician. This method produces interrater reliability concerns, steep learning curves, and likely misses important information that exists below the threshold of human detection. These challenges may be addressed with motion tracking and artificial intelligence technologies [12–18]. Contemporary advancements in computational capacity and machine learning offer such improvement towards a more objective, efficient, and automated assessment, especially useful for clinics with fewer resources and knowledgeable clinicians to effectively carry out and manage DBS implantation and adjustment.

The primary objective of this study was to improve the objectivity and automation of motor assessment in the operating room during motor mapping. This issue has been addressed in the past with traditional motion tracking software dependent on sensors or markers for object detection [19], which can be cumbersome, require expensive equipment, and additional setup. Thus, it is prohibitive for certain operating room (OR) environments. Recent developments in machine learning and markerless image tracking allow for simple setups ideal for the OR that require only video data. One program well-suited for this type of motion tracking is DeepLabCut (DLC), an open-source Python-based suite [20] used to track points of interest in video recordings. It has been used for collection of kinematic data across a myriad of organisms in diverse settings, including in neuroethological and human-based studies [21, 22]. Additionally, it is adaptable to low camera resolutions and variable light conditions [20–22]. This markerless approach exhibits acceptable or better effectiveness at motion tracking in human-based studies when compared to inertial and electromagnetic sensors [23] and infrared physical markers [24–27]. Several recent studies have employed DLC in clinical settings to track joints of the body, with evidence of reliability [25, 28], further bolstering its clinical applicability and utility. DLC has repeatedly demonstrated better performance and greater versatility in markerless label placement compared to other markerless methods like OpenPose and LEAP, which do not permit network retraining and comparatively have shallower, less robust networks [26, 27, 29, 30]. Pereira et al. (2022) recently augmented the functionality of LEAP as SLEAP for labeling and tracking in multi-animal experiments [31]. The open-source nature and low-cost implementation (only requiring a camera and computer setup) of DLC further elucidate its clinical utility, especially in non-Western and/or underfunded movement disorders centers [22, 32]. This machine learning-based pipeline could also prove useful for ruralized and under-resourced DBS clinics; employing an objective pipeline for movement identification and classification may alleviate concerns regarding clinician expertise and implantation efficacy, thereby improving quality of care [33]. Additional pioneering work has employed DLC in real-time [34–38], thereby demonstrating the potential for immediate feedback in an operating room setting.

Previous studies have employed two-dimensional pose estimation in conjunction with binary classification or principal component analysis to automatically extract features of gait aligning with clinical parameters and bradykinesia indicators to assess Parkinson’s disease severity [39–41]. Recent groups have demonstrated utility of machine learning and markerless pose estimation approaches for sit-to-stand gait analysis [17], classification of ataxia severity [18], and hand movements [42] diagnosis of a myriad of neurodevelopmental disorders, with promising results. Others have used less-robust decision tree, discriminant analysis, and nearest-neighbor machine learning algorithms to automatically score the severity of tremor in Parkinson’s disease patients [14, 15, 43]; as well as to facilitate DBS adjustment [44]. Recent groups have also added the ability to perform near real-time classification of human and animal movement using random forest classifiers [45]. However, to our understanding, the use of markerless pose estimation, deep neural networks, and support vector machines (SVM) for movement classification and optimal DBS placement is a novel application.

Here we use DLC to identify episodes of upper-body motor behaviors in patients with PD undergoing DBS implantation surgery, and subsequently distinguish these episodes using a binary classifier system. This is achieved by using markerless image tracking with DLC to follow body parts and extract episodes of two upper-body movements from initiation to termination as Euclidean distance epochs. To our knowledge, this study pioneers an approach to objectively identifying motor behaviors in the OR to assist clinicians’ judgment in functional MER for DBS electrode targeting. This approach is especially attractive as DLC’s robust output data can easily be refined into meaningful epochs and categorized by simple MATLAB commands. These results suggest that markerless tracking tools are a promising method for tracking kinematics in the OR and aiding in optimal DBS placement. This tool is particularly lucrative for under-resourced clinics; an investigation of non-Western deep-brain stimulation centers often in underdeveloped countries revealed that in some clinics, decisions on DBS candidacy and placement did not include a movement disorders neurologist (10.4%) or did not involve a committee whatsoever (53.5%) [46]. 33% of clinics did not employ a neurologist for DBS programming and 69% reported underutilization of DBS due to poor clinician knowledge [47]. The potential for this tool as a low-cost, automated method for identifying and evaluating motor behaviors in movement disorders has great utility in clinical settings with funding and staffing issues or ruralized difficulties that would otherwise be limited in offering DBS therapies.

## Materials & methods

### Study participants & enrollment

We collected intraoperative kinematic recordings from five subjects (5 male) recruited at the University of Colorado Anschutz Medical Campus through the Movement Disorders Center from the population of adult patients undergoing STN-targeted DBS surgery for treatment of PD. Given the demographic of patients within the Movement Disorders Center (MDC) at the medical campus, the predominant patient demographic centered around older-aged white men. In addition, other participants’ kinematic recordings, some of whom were women, yielded severely occluded videos. This was typically due to operating room dynamics and was unrelated to participant gender or racial identity. Surgical candidacy was assessed by a clinical panel composed of representatives from neuropsychology, neurology, neuroradiology, and neurosurgery, and determined based on well-established eligibility criteria [48]. Our study was carried out in accordance with the Colorado Multiple Institution Review Board (COMIRB; #17–1291) and Declaration of Helsinki with written informed consent obtained from all study subjects. For all subjects, written consent was received prior to surgical date and photocopies of their complete, signed consent were provided back to them.

### Intraoperative procedures

Following standard imaging-based stereotactic planning for trajectory and surgical targeting, intraoperative MER were used to locate the STN, using a standard approach detailed in prior work [49].

### Acquisition

**Fig 1:**
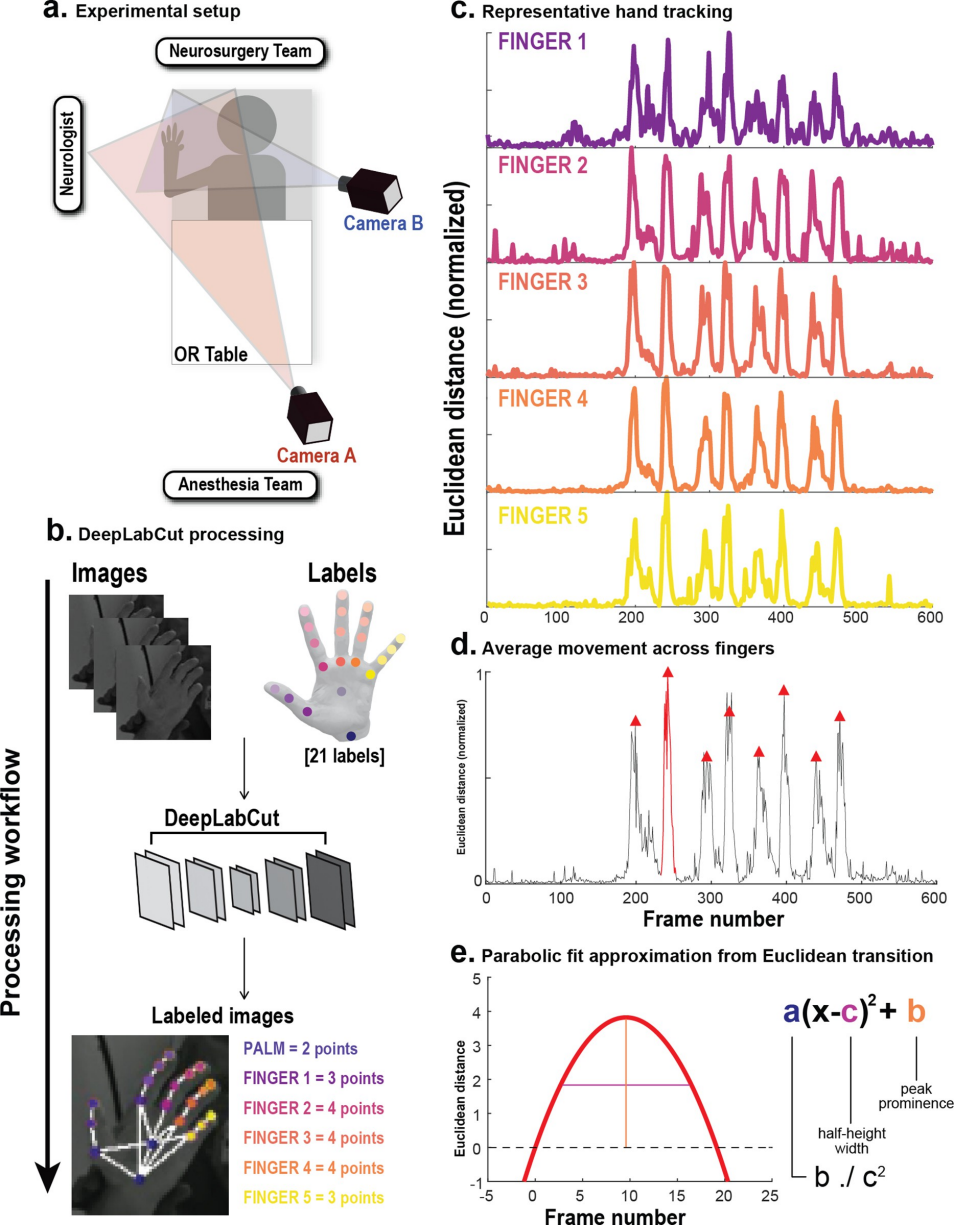
Processing workflow from DLC and post-hoc data analysis. (A) Setup of the two-camera recording system, highlighting all members of a typical neurosurgical team. (B) Simplified neural network consisting of inputted videos and labels. (C) Consolidation and smoothing of label trajectories into five groups, displayed on a normalized plot. Hand clench sample shown. (D) MATLAB’S *findpeaks* function employed to identify movement epochs. Half-height width and peak prominence extracted for each epoch. (E) Each epoch is fitted to a parabolic function with coefficients a, b, c to be added to the pipeline for movement type categorization.

Two FLIR cameras (USB 3.0 Blackfly) mounted on monopods (Avella A324D Aluminum 67 Inch Video Monopod) were oriented to capture motor testing (Fig 1A). To maximize the chances that an unobstructed view of the full range of motion be captured, one camera was positioned at the foot of the bed, and the other across the bed from where motor testing would occur. Cameras were connected to an independent laptop that triggered image capture using a custom Python script. A movement disorders neurologist assisted the patient in carrying out movements during video capture in a manner standard for the clinical evaluation of voluntary movements in assisting target localization for DBS.

### Processing

#### Kinematic data extraction

The two cameras were calibrated following data collection in the operating room. Calibration was performed to ensure that triangulation of video capture accurately labelled pertinent features [50]. This process used recordings of a checkerboard apparatus across the visual field, whereby corner detection was confirmed using a Python script. Relevant information was extracted, including camera angle (front and side) and video type. Individual frames were randomly selected to evaluate accuracy of checkerboard corner identification. This manual process ultimately confirmed the feature detector quality of DeepLabCut prior to kinematic data extraction. Inadequate frames were removed, as were paired frames from the other camera angle. Remaining frames were subjectively reviewed to further evaluate calibration. A mean reprojection pixel error threshold of <1 pixel was sought, which identifies the geometric error between a predicted versus actual point of interest in the visual field.

Following video acquisition, kinematic data were extracted post-hoc utilizing standard procedures developed as open-source tools within the DeepLabCut v2.2b6 suite [20]. Unique models were constructed using k-means clustering to isolate a subset of frames. Despite the wealth of evidence pointing to the effects of movement disorders on lower limb activity [1, 6, 9, 39, 51, 52], such aspects were not included in the present analysis due to the patient position in the operating room; a blanket was covering the patient during the awake DBS implantation and conducting analyses of lower limb kinematics would prove uncomfortable or unsafe for the patient. In addition, data were passively collected while the neurologist conducted their routine motor testing without explicit interaction from or coordination with the research recording to ensure unobtrusive collection and to test the performance of our approach on the often variable process of this intraoperative assessment. Twenty-one anatomical landmarks of the ventral and dorsal hand were manually labeled in each frame, including the base of the palm, center of the palm, metacarpophalangeal joints, proximal interphalangeal joints, distal interphalangeal joints, and the tips of all digits (Fig 1B). In general, manual labels must be applied for training of the network at a rate of about 100–200 frames [20], though we chose to label μ = 776 frames per network/patient. We chose to surpass the minimum number of frames required given the quantity of videos recorded per patient, the variations in camera quality and background, and to ensure that no network refinement was needed. Various parameters were adjusted prior to network training, including those responsible for training fraction and feature tracking [20]. The pretrained ResNet-50 network was used for all neural network trainings, which contains 50 iteratively trained layers in object identification and probability density mapping for accurate tracking, demonstrating efficacy given a small root mean squared error (3.09 ± 0.04) and accurate tracking on a subset of inputted data [20].

Various other parameters were adjusted to establish optimal memory allotment per training iteration, optimal filtering and smoothing, and optimal likelihood thresholds for feature tracking [20] (S1 Table). Models were trained until a plateau was reached in the network performance. The networks suggested proficient performance as indicated by >95% accuracy of labeled test frames. In instances where accuracy was below the >95% threshold, sessions were not considered for further evaluation. Under such standards, video samples per patient deemed viable increased given continued refinement of parameters and training iteration count, yielding a wealth of usable data. DeepLabCut’s native 2D median filtering was employed as a data cleaning measure to resolve discrepancies in pose estimations not due to occlusion [20]. No further network refinement was needed thereafter.

#### Movement epoch capture and processing

**Fig 2:**
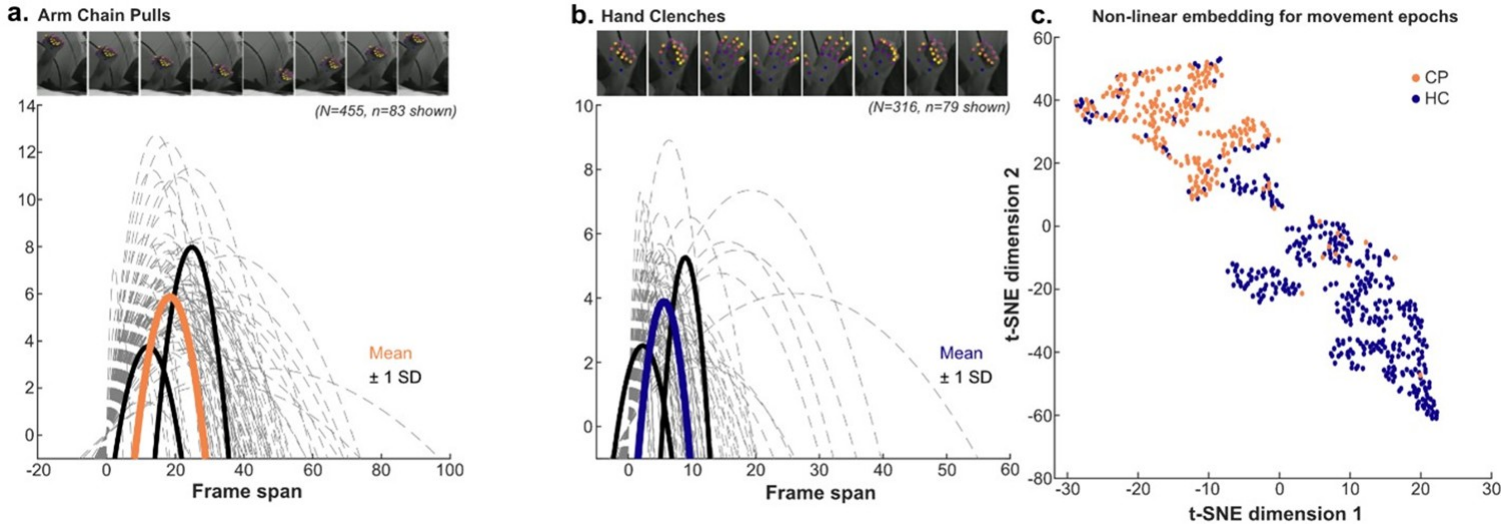
Movement epoch parabolic approximation and coefficient clustering. (A) Samples of parabolic fits for arm chain pulls displayed in light grey, alongside mean and standard deviation. (B) Samples of parabolic fits for hand clenches displayed in light grey, alongside mean and standard deviation. (C) Output of t-SNE (t-Distributed Stochastic Neighbor Embedding) to highlight distinct clusters of each movement type.

Data were then exported to MATLAB R2021a wherein kinematic metrics of each label including Euclidean distance, cosine similarity, velocity, and acceleration were calculated. We used Euclidean distance as the primary extrapolation of kinematic information for all post-hoc analyses; the inherent derivative accounted for spatial deflections and a priori insights on behavior could be extracted compared to other methods involving dimensionality reduction. Movements captured included chain pulls (CP) and hand clenches (HC). CP were defined by a starting position of 90° horizontal adduction and 90° external rotation of the glenohumeral joint, followed by repeated elbow extension and flexion, like the action of a latissimus pull down. HC were defined by repeated flexion and extension of the distal and proximal interphalangeal joints of the digits, like a clenching action. Plots of each labeled point were averaged across each digit and normalized. Palmar labels were omitted given little movement captured during HC. All putative examples of the two behaviors in question captured by a peak-identifying function in MATLAB were included in the pipeline. These plots were overlayed onto the original video recordings using a MATLAB script to verify that Euclidean distance epochs matched visually confirmed CP and HC movements as a ground-truth comparison (S1 Table). For epoch identification and extraction in MATLAB, ED plots were further averaged across the whole hand and smoothed, and various quality control measures were enacted to ensure only relevant movement epochs be identified (S1 Table). These epochs were fit to the parabolic function $-a{\left(x-c\right)}^{2}+b$, whose three coefficients defined clusters by which inputted active motor responses were categorized and identified. The coefficient “b” was peak prominence, and the coefficient “c” was half-height width. The “a” coefficient was computed as $\frac{b}{c^{2}}$ to complete the vertex form of the parabola (Fig 1D and 1E). The final table used in the following steps included these coefficients for each instance of movement (i.e., each epoch), and a column to code the ground-truth type of movement occurring. This was done for all movements, resulting in N = 771 total epochs (Fig 2A).

To combat errant movement epochs from rapidly successive movements (e.g., a movement hitch during a chain pull), a custom MATLAB script isolated complete episodes from motor initiation to termination. This was accomplished by identifying the intersection of x-values of each epoch’s peak at its half-height width and tracing in either direction for a minimum y-value before a change in slope direction. Provided that the minimum y-value was at or below a defined movement threshold, each movement epoch could be extracted as one complete motor event. These epochs could subsequently be fit to the parabolic equation as previously described.

#### Binary classifier system

**Fig 3:**
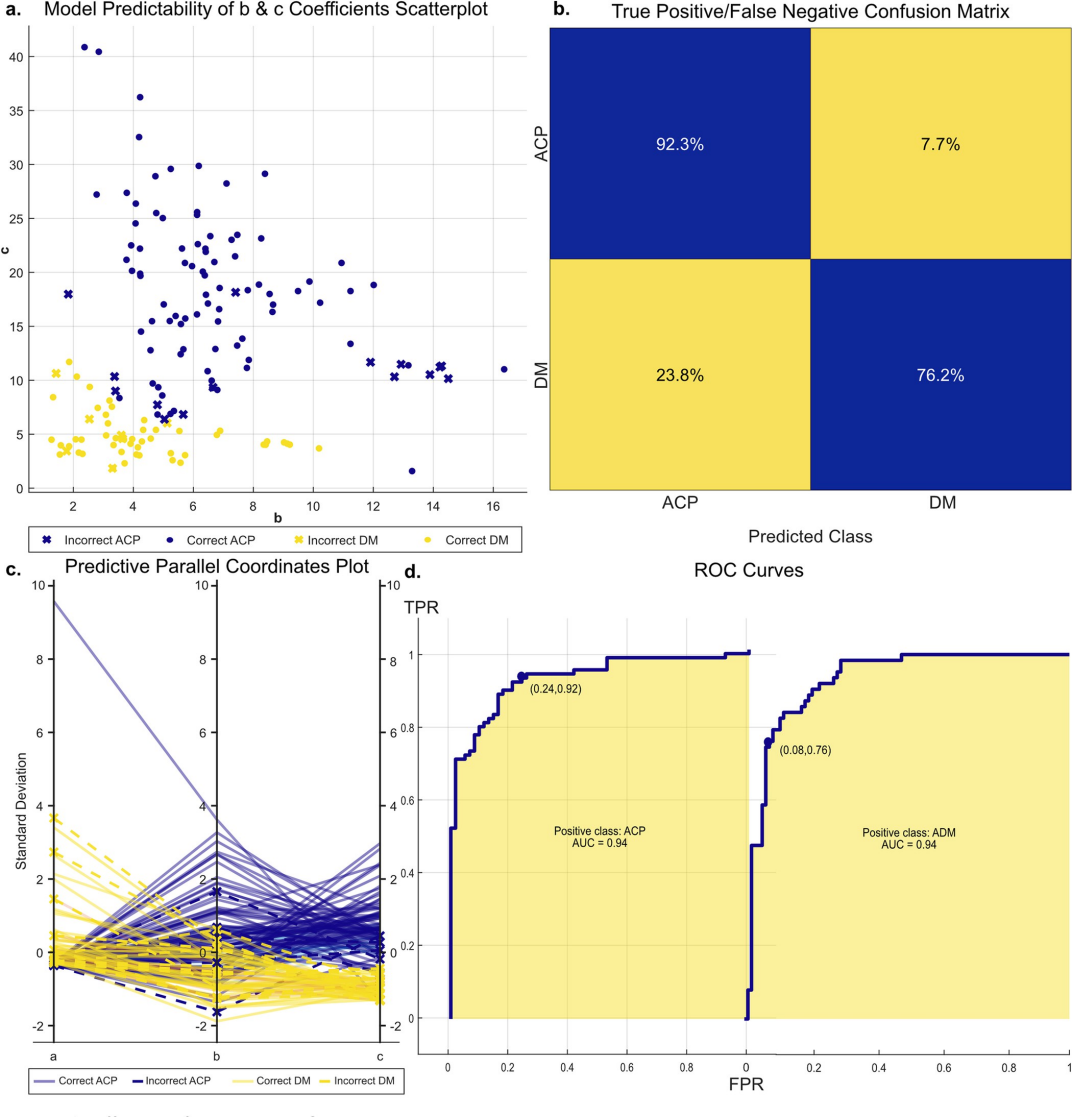
Efficacy of optimizable SVM. (A) Scatterplot of the movement clusters based on coefficients b and c of the parabolic fits, with arm chain pulls (CP) in blue and hand clenches (HC) in yellow. (B) True positive and false negative rates for predictive ability of the SVM as represented by a confusion matrix. (C) Parallel coordinates plot of the predictive ability of the model, highlighting the spread of coefficient values. (D) Receiver operating characteristic (ROC) curves between each class of the binary classifier.

We aimed to build a classifier to differentiate movements using the kinematic data derived from DLC in the OR. An optimizable support vector machine (SVM) model was built using MATLAB’s Machine Learning Toolbox. An optimizable SVM was chosen given its ability to continually update hyperparameters and yield the best model outcome with a subset of data (20% holdout). Hyperparameters such as kernel function, kernel scale, and box constraint level varied among models, whereas Bayesian optimization, iteration count, predictor variables (n = 3; parabola coefficients), and response classes (n = 2; CP and HC) were constant (Table 1). For the SVM model, Bayesian optimization was employed with the acquisition function “Expected improvement per second plus.” This approach asserts that expected improvement can be represented as:

$${EI(x,Q)=E}_{Q}[max(0,\mu Q(x_{best})-f(x))]$$

a cluster of acquisition functions that iteratively updates a Gaussian process model function [53]. *f*(x) is the Gaussian model, wherein x is a bounded domain that can be numerical or categorical, implying that varying results can emerge from *f*(x). For the expected improvement (*EI(x*, *Q)*) function, the expectation function within (E_Q_) is defined by maximum value between the prior mean (*represented as 0*) and the lowest value of the posterior mean distribution at location x (*represented as μQ(x*_*best*_*)*) subtracted by the Gaussian model. This Gaussian process model is also updated per second, meaning that there exists variability in the time expended to evaluate expected improvement depending on the x values in the function. This time-weighting is represented by

$$\frac{{EI}_{Q}(x)}{\mu_{s}(x)}$$

where the numerator represents a one-dimensional aspect of the expected improvement function (*EI*_*Q*_*(x)*) and the denominator is the posterior mean of the Gaussian process model (*μ*_*s*_*(x)*). Lastly, “plus” implies an iterative correction to the kernel function if overexploitation occurs. The optimizable SVM model repeats this expected improvement function to correct hyperparameters 30 times. The present model terminated with a quadratic kernel function (Table 1). The success of the SVM binary classifier performance was assessed using predictor variables, minimum classification error, predictive parallel coordinates, confusion matrices, and receiver operating characteristic (ROC) curves (Fig 3). The predictor variable scatterplot shed insight on the accuracy of the SVM model at classifying types of movement across the sample, using the “b” and “c” coefficients to demonstrate diversity of movement epochs. The parallel coordinates plot also showed the diversity of movement epochs, though represented as deviations from mean values, and showed SVM performance therein. The confusion matrix highlighted the true-positive and false-positive rates of movement classification, which was graphically represented as ROC curves to extrapolate on SVM performance by showing its diagnostic ability as thresholds varied.

**Table 1:** Optimizable Support Vector Machine (SVM) parameters and performance.

	Parameters and Values	
**Training Results**	Accuracy (Validation) Type	85.7% Holdout, 20%
	Total cost (Validation)	22
	Prediction speed	~22000 obs/second
	Training time	180.59 seconds
**Model Structure**	Response classes	2, including active hand clenches (HC) and arm chain pulls (CP)
	Predictors	3 (a, b, c coefficients)
	Observations	771
**Optimizer Options**	Optimizer	Bayesian optimization
	Acquisition function	Expected improvement per second plus
	Iterations	30
	Training time limit	False
**Optimized Hyperparameters**	Kernel function	Cubic
	Kernel scale	1
	Box constraint level	0.0010138
	Multiclass method	One-vs-One
	Standardize data	True
**Feature Selection**	All features (response classes) used in the model; principal component analysis (PCA) disabled	
**Misclassification Costs**	Default cost matrix	

Training results describe the performance of the binary classifier (SVM model) after 30 iterations of training. Model structure describes the input and output variables. Optimizer options describe the customizable optimizer and acquisition function types. Optimized hyperparameters describe the final model parameters after training.

## Results

### Video quality and DLC kinematic data extraction

We first assessed the quality of video data with respect to camera position and lighting conditions (Fig 1A). Following the calibration process [54], mean reprojection pixel error values ranged from 0.016–0.041, which was in line with the accepted threshold of <1 pixel [20], suggesting precise camera setup and calibration. In total, 3,164 frames were labeled (2.42% of 130,800 total frames; Table 2) in video recordings. Training was carried out until a plateau in Huber loss [20], yielding an iteration count of μ = 208,160 ± 13,730. This training duration resulted in 46.15–100.00% of videos per patient exhibiting >95% accuracy at tracking their motor behaviors (Table 2). On a subset (10%) of video recordings, the automated extraction script exhibited 80% accuracy at isolating movement epochs without manual intervention. 771 episodes of movement represented as parabolic functions (with duration μ = 0.44sec, SD = 0.29sec) were extracted from 98 video samples (20-120sec each) among five patients (57 ± 12 years of age, n = 5 Caucasian identity, with PD duration of 8 ± 2 years), including 455 chain pulls and 316 hand clenches (Fig 1E).

**Table 2:** Patient demographics and video/DeepLabCut descriptives.

	Demographics						
**Participants**	Sex			Male (n = 5), female (n = 0)			
	Age (in years)			57 ± 12			
	Racial identity			White/Caucasian (n = 5)			
	Years since PD dx			8 ± 2			
**Surgeries, Videos, & Training**		**Patient 1**	**Patient 2**		**Patient 3**	**Patient 4**	**Patient 5**
	Video count	26	26		36	10	16
	MRPE	0.018	0.03		0.041	0.016	0.017
	Percent of frames in model	5%	4.5%		1.85%	1.52%	3.57%
	Huber loss	0.0022	0.0021		0.0019	0.0022	0.0022
	Iteration count	195,200	204,800		195,700	220,900	224,200
	Epoch count	134	137		155	226	119

MRPE, mean reprojection pixel error; dx, diagnosis; PD, Parkinson’s disease; DeepLabCut parameters: MRPE, % of frames in model, Huber loss, and iteration count. Epoch count describes the number of complete movements captured per patient.

### Movement type identification pipeline performance

We next quantified the separability of the movement types; wherein distinct properties were observed between the parabola coefficients for CP compared to HC. Sampling a subset of these kinematic episodes revealed qualitative and quantitative differences between the apex height and peak half-height width (Fig 2A and 2B). Independent-samples t-tests revealed that peak prominence (coefficient b) of CP (*μ =* 6.1483, *SD =* 2.6024) differed significantly from HC (*μ =* 5.0619, *SD =* 2.8254; *t*(769) = 5.48997, p < .00001, two-tailed). The half-height width (coefficient c) had statistically-significant differences between CP (*μ =* 18.2025, *SD =* 7.7138) and HC (*μ =* 5.9578, *SD =* 3.8068; *t*(769) = 26.08718, p < .00001, two-tailed). Finally, the computed coefficient a logically demonstrated statistically-significant differences between CP (*μ =* 0.0643, *SD =* 0.3022) and HC (*μ =* 0.3004, *SD =* 0.3522; *t*(769) = -9.97051, p < .00001, two-tailed).

### Accuracy of SVM predictive model

With the eventual goal of real-time detection of classified active movements, we next determined whether movement types could be predicted based on these parabolic coefficients. We employed a support vector machine (SVM) model to distinguish between one of two response classes (Table 1). To train the model, 20.00% (n = 154 epochs) of the data were randomly withheld as input data to categorize the remaining 80% (n = 617). Following 30 iterations of training, taking 134 seconds, this model was able to predict the types of Euclidean distance movement epochs with 85.40% overall accuracy (Table 1). When comparing performance between movement types, the model exhibited slightly better ability at identifying arm chain pulls (92.30% accuracy) compared to hand clenches (76.20%), nonetheless indicative of high accuracy (Fig 3B). This performance was visualized by the scatterplot and parallel coordinates plot, which showed the SVM model’s hits and misses at categorizing movement types in the context of the wide variety of parabolic fits constituting the 771 epochs. Coefficient values generally ranged within five standard deviations of each respective average, highlighting the variability of movements that the model was tasked to categorize (Fig 3A and 3C). The ROC curves further demonstrated the predictive ability of the model; curves closer to the top-left corner indicated greater sensitivity and dominance of true-positives in correctly categorizing movement type (Fig 3D).

To ensure that this computational clustering approach was appropriately distinguishing between movements, we conducted supplementary reliability tests to ascertain the weight of each patient’s kinematic samples on the model performance. This was achieved by building pipelines consisting of four patients’ samples to predict those in the omitted dataset. This leave-one-out approach highlighted minimal variability in categorization accuracy, ranging from 91.50–94.50%. These values indicated that our pipeline was able to identify kinematic episodes without preference towards one case or another. This invariability and satisfactory performance of the model further demonstrated an adequate sample size with equal weighting therein.

## Discussion

Here we introduced an automated system that extracts and categorizes stereotyped upper limb movements captured with the markerless tracking Python-based suite DeepLabCut in the operating room during DBS surgery for aiding therapeutic targeting. Our findings demonstrated accuracy of the pipeline at identifying types of active motor behaviors in five patients undergoing DBS procedures for Parkinson’s disease. The model categorized arm chain pulls with better accuracy (92.30%) than hand clenches (76.20%). It is theorized that this discrepancy could be due to greater incidence of motion blur during rapidly successive movements, wherein the neural network’s peak confidence drops and its pose estimations are poorer [55]. By employing a leave-one-out approach to assess the relative weight of each patient’s set of exemplar movement episodes on the overall model, we yielded little variation in accuracy, thereby demonstrating a robust, generalizable dataset of movement episodes wherein no patient’s sample individually determined the overall SVM model performance accuracy.

These promising results hold significant clinical and rehabilitative implications. DBS treatment for advanced neurodegenerative disorders such as Parkinson’s disease often rely on localization and appropriate placement for optimal outcomes [2–6]. One disadvantage of this approach is the need for experienced clinicians to interpret the data. Our results suggest that markerless tracking tools are a good match for the challenges of this approach. Comparing markerless tracking to physical markers and sensors yields acceptable accuracy, and thus is a viable alternative approach with minimal setup [20–23, 26, 56]. Its independence from physical trackers or sensors permits seamless integration into the neurosurgical operating room where time is precious and sterile field must not be compromised, thereby alleviating the burden of maintaining cleanliness or continually replacing physical markers during surgery. The subjective assessment of Parkinson’s disease severity, denoted the Unified Parkinson’s Disease Rating Scale (MDS-UPDRS), only exhibits moderate reliability [57, 58]; this reliability is even poorer for evaluation of tremor, a hallmark of Parkinson’s disease [59]. Multiple studies elucidate the promising nature of objective means like markerless motion tracking and machine learning algorithms at augmenting the reliability of movement disorder symptom evaluation [12, 13, 15–18]. In turn, improving assessment reliability yields better patient outcomes. The present study adds to the current body of literature exploring this exciting avenue for improving Parkinson’s disease evaluation and treatment, including work using similar pose estimation and machine learning approaches for extracting features of gait, ataxia, bradykinesia, tremor severity, and various human movement classifiers [14, 17, 39–43]. We unprecedently add to this contemporary work by demonstrating initial steps at applying markerless pose estimation and simplistic machine learning algorithms to automatically assess upper limb movement during DBS localization and implantation.

Given the accuracy of our approach, markerless tracking technologies could be expanded beyond the operating room to objectively assess progression or severity of other neurodegenerative or neuromuscular disorders. For example, neurologists have employed DeepLabCut in post-stroke patients to conduct gait analyses with promising accuracy [60]. Our contributions could elaborate on this use by categorizing aspects of gait based on Euclidean distance movement epochs, thereby presenting an entirely objective option that performs well even in the absence of strictly-controlled room conditions required for videotaped observational gait analysis [25]. In pediatric patients, rehabilitation scientists have used a similar methodology to evaluate dyskinetic cerebral palsy symptoms, which could also be expanded upon by our findings [61].

Though powerful, this methodology does have limitations. Most notably, more patients are required to develop an increasingly robust dataset for continued refinement of the automated classifier system. In our model, only two active movements were included mainly due to occlusion of passive movements by clinicians’ hands or other objects in the visual field. The DeepLabCut algorithm cannot reliably approximate label locations during these passive movements, resulting in erroneous pose estimations that had to be discarded [55]. In addition, given the demographic of patients entering the MDC at the University of Colorado Anschutz Medical Campus, patients all tended to be white and of advancing age. There are presently no known studies that evaluate the performance of DeepLabCut across human demographic and intersectional lines (i.e., sex, race, size, etc.); however, training a new neural network per patient is certain to resolve this concern. DeepLabCut performance is also dependent on the quality of its training data, which is reliant on manual labelling of a subset of frames that can be time-consuming [20, 56]. We chose to dramatically increase the number of labeled frames per neural network compared to recommendations by [20] to augment quality of the networks and bypass additional network refinement. However, as few as 100–200 frames per network can be adequate, coupled with subsequent refinement [20]. These considerations are further exacerbated by the time required to train the neural networks even with GPU acceleration. Additionally, manual labelling can leave training data prone to operator error, thereby diminishing the standardizable nature of the output data. Generally, subjective review is advised to ensure the accuracy of markerless tracking and correct any discrepancies should they present.

Future work on this automated pipeline should aim to address these limitations. More data should be added to the current dataset by using markerless tracking and building deep neural networks on additional patients in the neurosurgical operating room, with a particular emphasis on passive and subtle movements. Including two movements is a promising first step, though future work should aim to capture and categorize more types of movements to represent the diverse nature of human kinematics as it applies to neurodegenerative disorders. Additionally, approaches should increase the resolution of extracted Euclidean distance epochs for processing and identification. Our current model employs two values drawn from parabolic fits to identify types of movement (with a third value calculated from the former two), whereas other methodologies like eigenface decomposition significantly increase the quantity of descriptive data [62]. By doing so, each movement epoch would exhibit a more robust numerical basis for categorization, which could augment our classifier system performance. Categorization may also be enhanced by including electrophysiological data, which could, in addition to kinematics, corroboratively improve both active and passive movement extraction and identification. Finally, the automated extraction script should be refined to confidently extract relevant Euclidean distance movement episodes without relying on manual intervention. We believe that upon continued refinement, the process may be used in real-time to entirely automate this pipeline, from DeepLabCut output to movement type categorization, as currently demonstrated in a myriad of animal models [34–38, 45]. This refinement will necessitate expansive collaboration with other movement disorders centers to amass large samples and generalizable models, as asserted by [12]. DeepLabCut’s model generalizability in rodents has been robustly demonstrated by [63], thereby elucidating the promising future developments of this automated pipeline. Regardless, the accuracy of markerless tracking at capturing active motor events and its efficient identification with only two parameters is highly indicative of clinical and rehabilitative utility. This technology could see rapid improvements and reliable integration in clinical and nonclinical settings across the world, especially in operating rooms limited by knowledge of and capacity to implant and evaluate DBS efficacy. Ruralized and non-Western under-resourced clinics would benefit greatly from this simple approach, as it is interpretable with minimal training. Thus, this methodology could augment accessibility and success of diagnostic and therapeutic approaches for individuals experiencing neurodegenerative, neuromuscular, or alike disorders [33, 46, 47].

## Supporting information

S1 Table DeepLabCut and MATLAB script parameters. (DOCX)
