# Supplementary material for: Automatic extraction of upper-limb kinematic activity using deep learning-based markerless tracking during deep brain stimulation implantation for Parkinson’s disease: A proof of concept study
Source: PLoS One. 2022 Oct 20;17(10):e0275490. doi: 10.1371/journal.pone.0275490 (PMC9584454; doi:10.1371/journal.pone.0275490)
Supplement: S1 Table — (DOCX) [file pone.0275490.s001.docx]

**Supplementary Material (S1). DeepLabCut and MATLAB Script Parameters**

| **Function** | **Parameters** |
| --- | --- |
| **DeepLabCut**  **v2.2b6** | - K-means extraction: A clustering algorithm that extracts a subset of frames for manual labelling in DeepLabCut. - Training fraction: Identifies the fraction of network data reserved for training versus testing. Set at 0.95 (95% reserved.) - Batch size: Establishes the computational memory allotted for each training iteration in the neural network. Set at 1 (one frame analyzed at a time.) - P-cutoff: Establishes the probability threshold for the neural network to positively label a point. Set at 0.06 for the small points of interest in our sample. - Positional distance threshold: Establishes the minimal acceptable distance between digital labels; is a greater value for smaller labels. Set at 17, raised from 15 after the neural network for patient 2. - Default augmenter: Defines the type of filtering and smoothing package used to correct skewed or missing labels. Set as imgaug and 2D median filtering. |
| **readFrame**  **(MATLAB r2021a)** | - Video reader: Reads .avi and .mp4 files containing patient kinematic recordings. - Linspace: Euclidean distance plot overlay in the video output spans the number of frames in each video recording (ranges from 600-2400 frames.) - Smooth data: Euclidean distance plot overlay in the video output is normalized and smoothed using the lowess function. |
| **findpeaks**  **(MATLAB r2021a)** | - Probability threshold: Based on p-cutoff and positional distance threshold, specifies the lowest probability for a movement epoch to be valid and extractable. Set at 0.65. - Minimum peak width: Establishes the smallest half-height width of Euclidean distance movement epochs to find and label as a peak. Set at 3. - Minimum peak prominence: Establishes the smallest prominence to find and label movement epochs as a peak. Set at 0.172, with extent annotations. - Minimum peak distance: Establishes the smallest separation (shortest duration of time) between successive peaks to avoid overlapping peaks or noise. Set at 20. |

Various parameters used throughout the movement epoch analysis pipeline. Describes parameter function and assigned values.
